# In search of clinically relevant parameters to monitor successful omalizumab therapy in allergic asthma 

**DOI:** 10.5414/ALX01377E

**Published:** 2019-09-01

**Authors:** M. Probst, A. Gogolka, M. Krüll, O. Noga

**Affiliations:** 1 Institute of Allergy and Asthma Research Berlin,; 2 Medical Clinical for Infectiology/Pulmonology, Charité – Universitätsmedizin Berlin

**Keywords:** omalizumab, allergic asthma, asthma treatment, airway resistance, anti-IgE

## Abstract

Background: Omalizumab is approved as add-on therapy for the treatment of severe uncontrolled allergic asthma. Increase in quality of life and decrease of exacerbations and hospital admission, as well as immunmodulatory effects have been described with omalizumab therapy. However, to date there are few parameters to monitor success and to evaluate the individual advantage of this therapy for the patient. Furthermore, no reliable parameter to predict response to treatment exists so far. The aim of this study was to define an easily applicable parameter for response to treatment with omalizumab. Method: 43 patients with allergic asthma were treated with omalizumab at a dose of at least 0,016 mg/kg/IgE every 4 weeks. Before, and 12 weeks after initiation of therapy, bodyplethysmography including airway resistance was performed. Efficacy of treatment was judged by the attending physician on the basis of a five point chart. Furthermore, a differential blood count was performed before, and 12 weeks after initiation of treatment. Total and specific IgE against all relevant antigens were determined before start of therapy. Results: Airway resistance in patients with response to treatment with omalizumab (responders) was significantly decreased in comparison to patients without clinical benefit (non-responder). The number of eosinophil granulocytes in the peripheral blood was decreased in both groups without significant difference. Response to therapy was associated with younger age and lower levels of specific IgE against the allergen with the highest sIgE-level (seasonal and perennial), but not with the sIgE level of the perennial allergens in general. Conclusion: Measurement of airway resistance might be an additional parameter for monitoring response to therapy with omalizumab. High specific IgE levels, for both perennial and concomitant seasonal allergens as well as increasing age, seem to predict less favorable treatment outcomes.

**German version published in Allergologie, Vol. 34, No. 6/2011, pp. 299-306**

## Introduction 

Allergic asthma is an inflammatory airway disease and immunoglobulin E (IgE) plays a key role in the induction and maintenance of the inflammation. The prevalence of this chronic disease is very high and many patients are treated insufficiently [1]. Omalizumab – a recombinant humanized monoclonal anti-IgE-antibody – has been included in the GINA guidelines as a treatment for difficult-to-control bronchial asthma [2]. Its mechanism of action has not been used in the therapy of allergic asthma before. Circulating IgE is eliminated by the binding of omalizumab to the binding site of the high-affinity FceRI receptor on the IgE antibody, which is the reason why omalizumab is not anaphylactogenic. Furthermore, omalizumab induces the downregulation of the FceRI receptor on basophils and mast cells which potentiates the IgE complexing effect [3, 4]. In patients with asthma, omalizumab attenuates the early and late phases of an allergic reaction to inhaled allergens. In addition, several anti-inflammatory effects that possibly contribute to the clinical efficacy of omalizumab have been observed [6, 7, 8]. 

A number of clinical trials were able to demonstrate the efficacy of omalizumab treatment in patients with allergic asthma [9, 10, 11, 12]. It was shown that omalizumab therapy significantly improved quality of life and reduced the number of severe asthmatic exacerbations that frequently lead to hospitalization and admittance to emergency wards [12, 13]. 

Symptom improvement could, however, not be achieved in all patients. In a large number of evaluated patients the response rate was 61% [14]. 

It is difficult to predict whether or not a patient will respond to omalizumab treatment because the key parameters that point to clinical improvement in studies do not allow researchers to distinguish between responders and non-responders. Further parameters that show a significant effect under omalizumab therapy are either experimental and aim at measuring the role of IgE in the allergic inflammation process or they are not adequate for the evaluation of individual efficacy. In an evaluation of various variables of clinical benefit, the investigator’s global evaluation of treatment effectiveness (IGETE) was shown to be the most important parameter with regard to the efficacy of omalizumab [14]. The high costs of omalizumab therapy further support the need for parameters that can easily be applied in clinical practice. 

The reasons why only 61% of patients respond to omalizumab remain unknown. It could be assumed that in non-responders the allergy is not an important trigger of the inflammation or that the elimination of free IgE is insufficient with regard to the release of mediators. 

The aim of our study was to evaluate bodyplethysmography and differential blood count as parameters for the monitoring of the efficacy of omalizumab therapy. Furthermore, we investigated the role of specific IgE levels against various allergens as predictive parameters. 

## Methods 

### Study design 

We performed a retrospective analysis of 44 patients with allergic bronchial asthma who received omalizumab therapy. All patients had allergic asthma with a positive skin prick test to a perennial allergen (Dermatophagoides farinae, Dermatophagoides pteronyssinus, cat or dog) and according allergen-specific IgE (sIgE) (ImmunoCAP, Phadia, Uppsala, Sweden). Seasonal allergens were assessed in the same way. All patients were treated with an inhaled glucocorticoid and showed reversibility of the forced expiratory volume in 1 second (FEV_1_) within 30 minutes after inhalation of 200 mg salbutamol of more than 12% as compared to the baseline value. Omalizumab was administered subcutaneously every 2 – 4 weeks. The dose was adjusted to the patient’s body weight and total IgE at screening in order to reach a minimum dose of 0.016 mg/kg/IgE every 4 weeks. Therapy benefit was evaluated after 12 – 16 weeks of therapy. 

### Bodyplethysmography 

Bodyplethysmography was carried out between 8 a.m. and 2 p.m. by qualified staff according to the recommendations of ERS/ATS [15, 16]. 

### Peripheral blood count/eosinophil granulocytes 

Peripheral blood count/eosinophil granulocytes were determined using automated detection methods. Blood was drawn by peripheral vein puncture. 

### Investigator’s global evaluation of treatment effectiveness (IGETE) 

The treatment effectiveness was evaluated on a 5-point scale: –2 = pronounced aggravation of symptoms; –1 = aggravation of symptoms; 0 = no change; 1 = symptom improvement; 2 = pronounced symptom improvement. Patients with a value of 0 or below were classified as non-responders; responders reached a value of 1 or 2. 

### IgE determination 

Total IgE and specific IgE were determined using the CAP system (Phadia, Uppsala, Sweden). 

### Statistical analysis 

Inter-group differences were compared with the Mann-Whitney-U test. A p-value < 0.05 was considered statistically significant. If not stated otherwise, all tests compared the data of the treatment groups as percentages of change from the baseline value to therapy week 12. For comparisons within-group (before and after initiation of treatment) the Wilcoxon test was used. A p-value < 0.05 was considered statistically significant. 

## Results 

### Patients 

Between 1998 and 2006, a total of 44 patients were treated with omalizumab. The patient characteristics are presented in Table 1. 26 patients (60.5%) were classified as responders (IGETE 1 or 2) and 17 (39.5%) as non-responders (IGETE 0 to –2). It was shown that younger patients responded significantly better to the treatment (Table 1). 

### Airway resistance as a parameter of the response to treatment 

**Figure 1.**
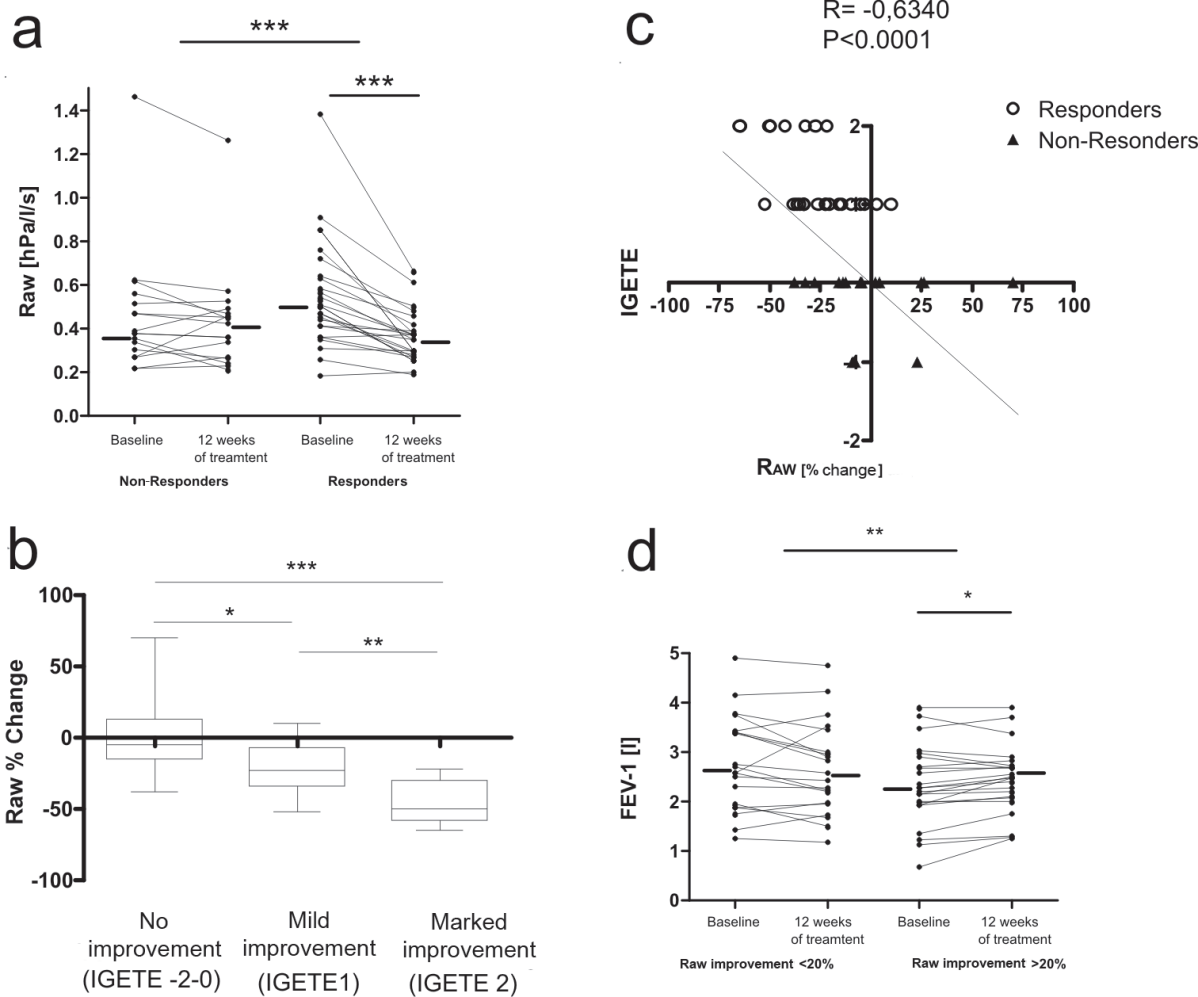
a: Individualized presentation of baseline values and values after 12 weeks of omalizumab therapy. The group of responders (n = 26) showed a significant reduction of airway resistance as compared to the group of non-responders (n = 17). In the group of responders, the reduction of airway resistance measured before, and 12 weeks after initiation of therapy, was significant. The horizontal lines represent the median value. *p < 0.05; **p < 0.01; ***p < 0.005. b: Change of airway resistance (from baseline value) as a function of the level of clinical response. *p < 0.05; **p < 0.01. c: Correlation between the change of airway resistance and the level of clinical response. d: FEV: Individualized presentation of baseline values and values after 12 weeks of omalizumab therapy. Response to therapy is defined as ³ 20% reduction of airway resistance. In the group of responders, a significant reduction of FEV as compared to the group without reduction of airway resistance was observed. The horizontal lines represent the median value. *p < 0.05; **p < 0.01; ***p < 0.005.

The comparison of airway resistance (R_aw_) before the start of therapy showed a tendency towards higher values in the group of responders which did not, however, not reach statistical significance. Under omalizumab therapy, a highly significant reduction of airway resistance (p < 0.001) was observed in responders as compared to non-responders. The median reduction of airway resistance in the group of responders was 22.4% of the baseline value. The evaluation of the individual airway resistance values before, and 12 weeks after initiation of therapy also show significantly lower values during therapy, in responders, while in non-responders no difference could be observed (p < 0.001) (Figure 1a). The improvement of airway resistance corresponded to a clinical improvement during therapy (Figure 1b). There was a strong correlation between the reduction of airway resistance and clinical evaluation (r –0.634) (Figure 1c). A change of FEV_1_ under therapy could, however, not be detected in either group. On the other hand, if the classification as a responder was defined by the reduction of airway resistance, a significant improvement of FEV_1_ could be observed in this subgroup (Figure 1d). 

The subgroup analysis of patients receiving placebo (data published earlier) did not demonstrate a significant change of airway resistance before and after initiation of therapy (0.4 kP/s*l bis 0.42 kP/s*l). The number of responders among the placebo-treated patients was low (3 of 18 patients; 16.67%) [8]. 

### Peripheral eosinophil granulocytes did not correlate with the response to treatment 

**Figure 2.**
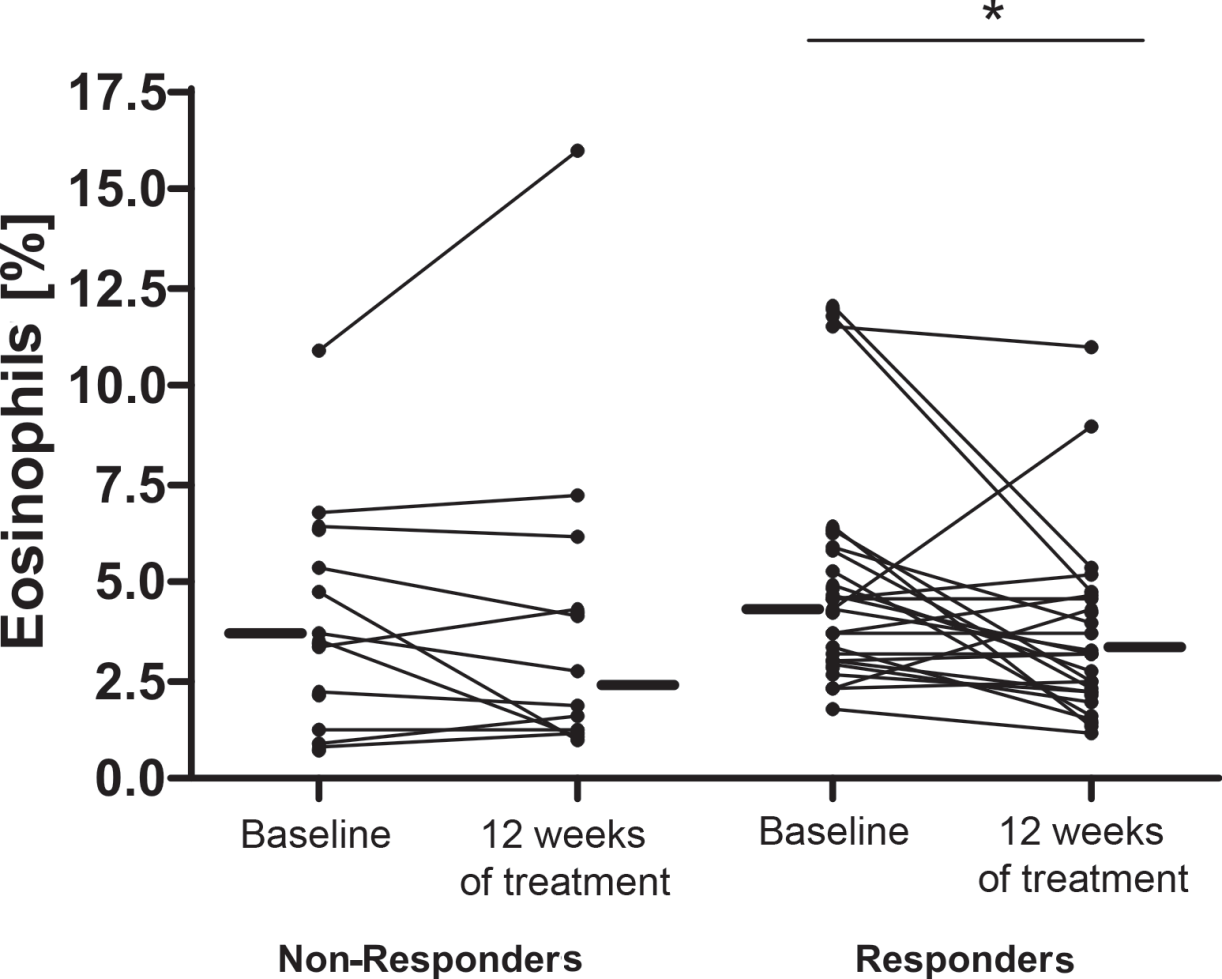
Change of eosinophil granulocytes (in percent of leukocytes) in the peripheral blood in the responding and non-responding group.

The comparison of responders and non-responders with regard to blood eosinophilia showed a slight reduction in both groups. In the group of responders this reduction was statistically significant (p < 0.05), but no significant difference between both groups could be observed (Figure 2). 

### The maximum specific IgE, but not the perennial allergen-specific IgE, is a predictor for good response to anti-IgE-therapy 

**Figure 3.**
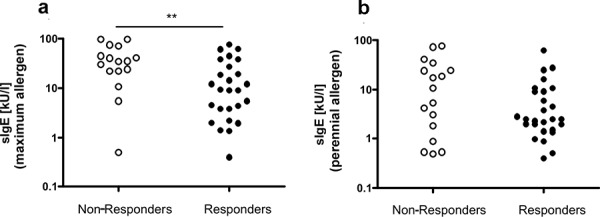
a: Individualized presentation of specific IgE against the perennial allergen in patients with good and bad response to anti-IgE therapy. b: Individualized presentation of specific IgE against the perennial allergen with maximum sIgE values (seasonal or perennial) in patients with good and bad response to anti-IgE therapy.

The comparison of perennial sIgE shows higher values in non-responders, without the difference being statistically significant. The comparison of the maximum sIgE-value (perennial or seasonal allergen) demonstrates significantly higher values in the non-responders (Figure 3). 

## Discussion 

Our study showed that airway resistance is an adequate parameter for the evaluation of a positive response to omalizumab treatment. Airway resistance is easy to measure and can be determined in routine practice. Due to the low number of cases in our study, we cannot determine the exact value of airway resistance decrease that would allow for the identification of responders. The mean decrease of airway resistance was 22.4%. There was a strong correlation with the results of the IGETE scoring. This means that the measurement of airway resistance can be an independent parameter for the evaluation of response to omalizumab treatment. Patients in whom an adequate reduction of airway resistance is not reached, even after repeated measurements, should be critically re-evaluated with regard to a possible benefit of omalizumab therapy. In addition to the well-established parameters of “quality of life”, “number of exacerbations”, and “necessary medication”, airway resistance can be a further useful parameter for evaluating whether omalizumab therapy should be continued or not. Hopefully, the standardized measurement of airway resistance under omalizumab therapy will generate more data in the future so that an adequate cut-off value for the reduction of airway resistance can be defined. The fact that the clinical response can be documented by an objective and sensitive marker of airway obstruction seems to be the logical consequence. If the clinical response is evaluated using the decrease of airway resistance, a significant improvement of FEV_1_ can also be observed. This might also explain the fact that in individual patients marked increases of FEV_1 _have been observed, while for the total study population no homogeneous improvement could be shown [17]. 

Younger age is associated with a better response to treatment. This could be due to the impact of allergies on the underlying inflammation and is generally expected. But this does not mean that older patients could not benefit from anti-IgE therapy. In principle, no age group should be excluded from therapy; nevertheless, the relevance of a sensitization for airway obstruction should be critically evaluated and other diseases – particularly COPD – should be excluded. 

The aspect of measured specific IgE levels seems to become more and more important in achieving an effective suppression of the cellular release of mediators. In this context, our data show that in addition to the specific IgE against the perennial allergen – which needs to be present to justify anti-IgE therapy – the maximum specific IgE value also seems to be of major importance. Furthermore, a routine measurement of free IgE would be desirable for the future. 

In our study, the determination of eosinophil granulocytes in the blood did not represent a further instrument for the evaluation of a patient’s response to anti-IgE therapy, although other data suggest that a reduction of IgE influences the circulating and tissue eosinophil granulocytes [6, 7, 8, 18]. The comparison of responders and non-responders with regard to the reduction of circulating eosinophils shows that the decrease in responders is only of low statistical significance. In the group of non-responders, a mild reduction could also be detected so that no significant difference between both groups was seen. The described reduction of eosinophil granulocytes does not seem to reflect any acute clinical effect. This assumption is supported by results from the anti-interleukin-5 studies, where a marked reduction of eosinophil granulocytes did not lead to any significant clinical effect [19]. As suggested by Kariyawasam and Robinson [20], eosinophil granulocytes do seem to play a role in long-term airway remodeling and long-term studies will be necessary in the future. 

In conclusion, we would suggest the evaluation of airway resistance in patients undergoing omalizumab therapy as well as the determination of the spectrum of perennial and seasonal allergens. Further data from larger study populations are necessary to make a final recommendation. 

## Conflict of interest 

The authors declare that with regard to this manuscript no conflict of interest was present. 

**Table 1.** Patient characteristics.

**Parameter**	**Baseline**		**12 weeks of therapy**	
44 patients 17 male 27 female	Non-responders	Responders	Non-responders	Responders
Age (n)	53 (23 – 72)	39^+^ (19 – 56)	53 (23 – 72)	39^+^ (19 – 56)
R_aw_ (kPa*s/l)	0.38 (0.22 – 1.46)	0.50 (0.18 – 1.38)	0.42 (0.21 – 1.26)	0.35***^, ##^ (0.19 – 0.66)
FEV_1_ (l)	2.56 (1.24 – 4.92)	2.37 (0.69 – 4.16)	2.36 (1.32 – 4.76)	2.52 (1.19 – 2.24)
Specific IgE perennial (kU/l)	10.7 (0.4 – 76.2)	2.5 (0.4 – 61.8)		
Specific IgE maximum value (kU/l)	35 (0.4 – 99)	9.17^++^ (0.4 – 78.1)		

^+^p < 0.05 (responders vs. non-responders), ^++^p < 0.01 (responders vs. non-responders), ***p < 0.01 (before vs. during therapy), ^##^p < 0.05 (responder vs. non-responders during therapy).
